# Seasonal variations drive microbial community structure and nitrogen cycling in sediments of tributary pumping station forebays

**DOI:** 10.1128/spectrum.03047-25

**Published:** 2026-05-11

**Authors:** Xiaofu Wei, Weiwei Song, Siyuan Li

**Affiliations:** 1 Institute of Water Science and Technology, Hohai University 12462, Nanjing, China; 2 National Engineering Research Center of Water Resources Efficient Utilization and Engineering Safety, Hohai University 12462, Nanjing, China; 3 College of Hydrology and Water Resources, Hohai University 12462, Nanjing, China; 4 College of Water Conservancy and Hydropower Engineering, Hohai University 12462, Nanjing, China; University of Mississippi, University, Mississippi, USA

**Keywords:** 16S rRNA and metagenomic sequencing, denitrification, functional genes, network analysis, PLS-PM model

## Abstract

Discharge from tributary pumping stations often impacts mainstream water quality, yet microbial communities and nitrogen metabolism in pumping station forebays remain poorly understood. Therefore, this study investigated the microbial community structure and nitrogen cycling mechanisms in sediments of tributary pumping station forebays within the Qinhuai River Basin using 16S rRNA and metagenomic sequencing. Results showed significant seasonal variations in the diversity and structure of sediment microbial communities, with higher diversity in spring than in winter. Genes associated with denitrification (e.g., *narG*, *nirS*, and *nosZ*) showed the highest abundance, suggesting that denitrification may be a key nitrogen transformation pathway. Co-occurrence network analysis revealed tighter associations between microbial taxa and nitrogen-cycling genes in spring, indicating more complex potential interactions during this season. The shift of network hubs across seasons suggested a seasonal succession of potential core functions related to nitrogen cycling. Redundancy analysis revealed that nitrate nitrogen (NO_3_^−^-N), water temperature (WT), and ammonium nitrogen (NH_4_^+^-N) were the factors most strongly associated with microbial community variation, with WT showing the strongest association with functional gene distribution. Partial least squares path modeling revealed that seasonal variation had a significant positive association with denitrification gene abundance and a significant negative association with genes related to assimilatory nitrate reduction to ammonium and anaerobic ammonium oxidation. These findings improve our understanding of microbially mediated nitrogen cycling in pumping station forebays and provide a scientific basis for water quality management in river networks influenced by pumping station drainage.

**IMPORTANCE:**

This study is important because it reveals that pumping stations, which are key infrastructure in managed river systems, are not just hydraulic structures but dynamic bioreactors where microbial communities actively transform nitrogen. By demonstrating seasonal variations in microbial diversity and revealing a high denitrification potential, the research provides a mechanistic understanding of how nitrogen pollution is naturally mitigated in these engineered environments. Crucially, it pinpoints temperature as a primary regulator of these microbial functions. These insights allow water managers to proactively optimize pumping operations and design interventions that harness microbial activity, ultimately protecting downstream water quality from nutrient pollution in a changing climate.

## INTRODUCTION

Rivers play a key role in maintaining aquatic ecosystem diversity and regulating global nutrient cycles (e.g., the nitrogen cycle) (1). However, their ecological functions are often compromised by anthropogenic infrastructures, among which pumping station forebays, transitional water bodies connecting drainage systems and receiving rivers, are of particular concern. Tributary pumping station forebays represent a unique and critical case. Unlike lakes characterized by stable hydrology and endogenous pollution or estuaries dominated by tidal forces and large-scale inputs, these forebays receive highly variable, pulsed discharges of stormwater-sewage mixtures from urban runoff. Furthermore, compared to non-tributary forebays, which handle larger, more regulated flows, they experience shorter hydraulic retention times (hours to a day) and frequent fluctuations driven by pumping and rainfall. Consequently, their internal dynamics can influence downstream water quality and ecosystem functioning. In combined or mixed sewer systems, these polluted mixtures are temporarily retained and enriched with nutrients, organic matter, and other pollutants in the forebay, disrupting the aquatic balance, altering microbial communities, and interfering with nitrogen metabolism. Their eventual discharge into receiving rivers causes acute issues such as elevated ammonium nitrogen and reduced dissolved oxygen (DO) (2). Thus, understanding microbial community composition and nitrogen transformation processes in tributary pumping station forebays is important for improving the management of nitrogen loading and water quality at this critical basin juncture.

Microorganisms are core drivers of biogeochemical cycles and ecosystem energy flow, playing vital roles in maintaining water quality, nutrient cycling, and ecological stability (3). Consequently, they have been extensively studied. Research across various aquatic ecosystems indicates that microbial communities are highly sensitive to external environmental changes, which significantly alter their structure and function (4–6). Crucially, studies in large-scale engineered water systems, such as China’s South-to-North Water Diversion Project, a major inter-basin water transfer scheme, have demonstrated that microbial communities (comprising bacteria, archaea, and fungi) are primarily driven by factors, including nitrate nitrogen (NO_3_^−^-N), nitrite nitrogen (NO_2_^−^-N), phosphorus forms, and water temperature (WT) (7, 8). Similarly, research in large, regulated reservoirs, such as the Danjiangkou Reservoir, has identified WT, DO, nitrogen species, and total dissolved solids as key drivers of planktonic microbial communities (9). These studies illustrate the key roles of environmental factors in shaping microbial communities in human-regulated aquatic systems. However, compared with these large engineered systems, the environmental drivers of sediment microorganisms in tributary pumping station forebays, which are smaller in scale yet also strongly affected by human regulation, remain poorly understood. Methods such as redundancy analysis (RDA) and structural equation modeling, which are widely employed to quantify correlations between environmental factors and microbial communities (10–12), were therefore used in this study to address this knowledge gap regarding the environmental drivers of sediment microorganisms in tributary pumping station forebays.

The nitrogen cycle is one of the most important biogeochemical cycles in river ecosystems (13). Microbially mediated nitrogen transformation involves multiple interconnected pathways, including nitrification, denitrification, anaerobic ammonium oxidation (anammox), dissimilatory nitrate reduction to ammonium (DNRA), and assimilatory nitrate reduction to ammonium (ANRA) (14, 15). These processes regulate nitrogen removal, retention, and recycling in aquatic environments (16–18). In riverine sediments, the relative importance of these pathways is influenced by environmental conditions such as temperature, dissolved oxygen, nutrient availability, and organic carbon content (19, 20). Seasonal variations alter these environmental conditions and consequently lead to shifts in nitrogen transformation pathways and corresponding differences in the abundance of genes involved in nitrogen metabolism (21–23). Previous studies on sediment nitrogen transformations have been extensively conducted in various aquatic systems, including lakes, estuaries, and wetlands (24–26). However, engineered aquatic systems such as pumping station forebays often receive stormwater and combined sewage, which are temporarily stored and subsequently discharged into the main river channel, potentially contributing to river water quality deterioration. Despite their important role in regulating nutrient inputs to receiving waters, few studies have investigated changes in the microbial community structure of pumping station forebay ecosystems or the seasonal dynamics of nitrogen transformation processes in these environments. Consequently, the microbial mechanisms driving nitrogen transformation in such systems remain poorly understood.

In the present study, we focused on the Qinhuai River in Nanjing, a typical urban waterway influenced by multiple drainage pumping stations along its banks. We investigated sediments from the forebay of Qinhuai River pumping station, integrating 16S rRNA gene sequencing with high-throughput metagenomic sequencing to explore how seasonal variations affect the microbial community structure and nitrogen metabolic potential. This study aims to address the following scientific questions: (i) how do the microbial community composition and the genetic potential of key nitrogen-cycling processes shift between seasons in response to changes in fundamental water quality parameters (e.g., WT, DO, and nitrogen concentrations)? (ii) Which specific abiotic factors (e.g., WT and NO_3_^−^-N) and biotic factors (e.g., microbial network properties) are the primary drivers of these seasonal variations? (iii) How do the combined effects of these seasonal abiotic and biotic factors influence the nitrogen metabolic potential, and what are the functional implications (e.g., network complexity and stability) for the nitrogen cycle in this engineered ecosystem?

## MATERIALS AND METHODS

### Study area and sampling design

The Qinhuai River, located in Nanjing, Jiangsu Province (31°35′–32°07′N, 118°43′–119°18′E), is a major right-bank tributary in the lower reaches of the Yangtze River. Numerous drainage pumping stations were situated along the upstream and downstream riverbanks. The river basin has a typical northern subtropical humid climate, characterized by distinct seasons and abundant rainfall. The average annual precipitation is approximately 1,050 mm, with most rainfall concentrated from June to September.

**Fig 1:**
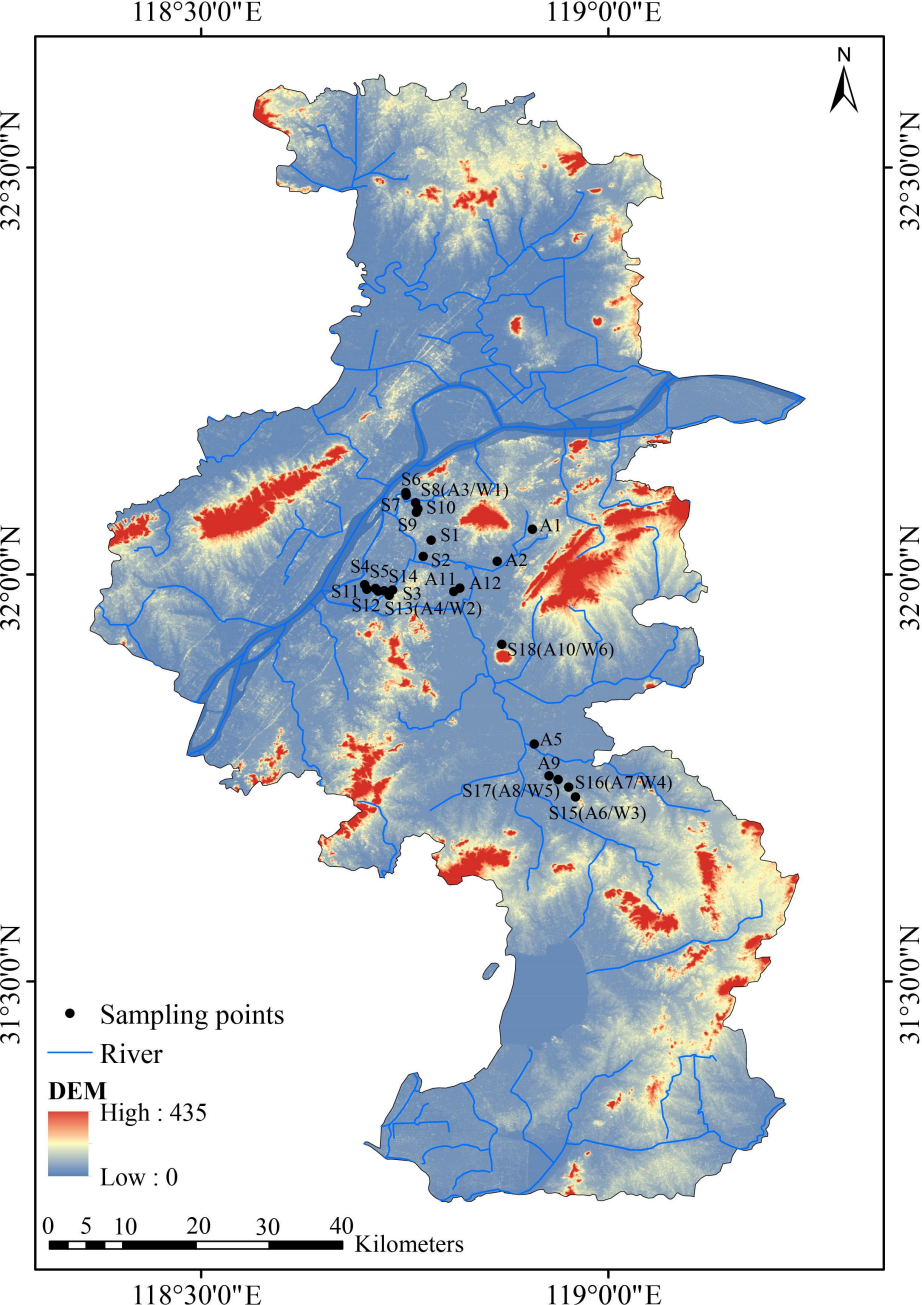
Study area and sampling point location. S, spring; A, autumn; and W, winter.

As shown in Fig. 1, sampling sites were established at the forebays of 24 tributary pumping stations along the Qinhuai River. At each site, paired surface water and sediment samples were collected simultaneously; in total, 36 water and sediment samples were collected during the three sampling events in April (spring), September (autumn), and December (winter) of 2024. The water samples were used for physicochemical analysis, whereas the sediment samples were designated for microbial community analysis. Due to restrictions on pumping station management, seasonal sampling was limited to 18 sites in spring, 12 in autumn, and 6 in winter. The specific sites sampled in each season are detailed in Table 1. At each site, surface water was collected approximately 0.5 m below the water surface using a plexiglass water sampler. One liter of water was collected in triplicate and then mixed to form a composite sample to minimize small-scale variability and obtain a representative sample of the water body at each site. Water samples were stored at 4°C and transported to the laboratory for physicochemical analysis. For sediment sampling, a stainless steel grab sampler (model: CN-100, manufactured by Guangzhou Ruibin Technology Co., Ltd., China) was used to collect sediment from the top 0–5 cm layer. Samples were taken at 1 m intervals around the center of each sampling site, in triplicate. After removing large impurities, the three subsamples were homogenized into one composite sediment sample to minimize the influence of small-scale spatial heterogeneity and ensure that the sample represented the average microbial community composition of the site’s surface sediment. All samples were placed in sterile polyethylene bags, kept on ice during transport to the laboratory, and subsequently stored at −80°C until DNA extraction.

**TABLE 1:** Sampling site locations

Sample ID	Season	Site	Longitude	Latitude
S1	Spring	Site 1	118.7613	32.01952
S2	Spring	Site 2	118.7518	31.99954
S3	Spring	Site 3	118.7139	31.95822
S4	Spring	Site 4	118.68	31.96466
S5	Spring	Site 5	118.6932	31.95982
S6	Spring	Site 6	118.7304	32.0776
S7	Spring	Site 7	118.7307	32.0746
S8	Spring	Site 8	118.7423	32.0652
S9	Spring	Site 9	118.7434	32.0536
S10	Spring	Site 10	118.7452	32.0579
S11	Spring	Site 11	118.6823	31.9592
S12	Spring	Site 12	118.6966	31.95683
S13	Spring	Site 13	118.7097	31.95161
S14	Spring	Site 14	118.7039	31.95723
S15	Spring	Site 15	118.9387	31.704
S16	Spring	Site 16	118.9304	31.7157
S17	Spring	Site 17	118.9172	31.72507
S18	Spring	Site 18	118.8482	31.89145
W1	Winter	Site 8	118.7423	32.0652
W2	Winter	Site 13	118.7097	31.95161
W3	Winter	Site 15	118.9387	31.704
W4	Winter	Site 16	118.9304	31.7157
W5	Winter	Site 17	118.9172	31.72507
W6	Winter	Site 18	118.8482	31.89145
A1	Autumn	Site 19	118.8856	32.03278
A2	Autumn	Site 20	118.8426	31.9932
A3	Autumn	Site 8	118.7423	32.0652
A4	Autumn	Site 13	118.7097	31.95161
A5	Autumn	Site 21	118.8882	31.76896
A6	Autumn	Site 15	118.9387	31.704
A7	Autumn	Site 16	118.9304	31.7157
A8	Autumn	Site 17	118.9172	31.72507
A9	Autumn	Site 22	118.9061	31.72999
A10	Autumn	Site 18	118.8482	31.89145
A11	Autumn	Site 23	118.7891	31.95626
A12	Autumn	Site 24	118.7965	31.96029

### Physical and chemical analyses

On-site physical and chemical indicators, including WT, pH, and DO, were determined using a portable multi-parameter water quality analyzer. For dissolved nutrient analysis (ammonia nitrogen [NH_4_^+^-N], NO_3_^−^-N, and NO_2_^−^-N), water samples were filtered through a 0.22 μm membrane filter to remove particulate matter, whereas unfiltered water samples were used for the determination of total nitrogen (TN), chemical oxygen demand (COD), and permanganate index (COD_Mn_). All analyses were conducted following standard methods in compliance with Chinese national standards (GB3838-2002).

### DNA extraction, sequencing, and analysis

Genomic DNA was extracted from 0.5 g of the frozen sediment samples using the FastDNA SPIN Kit (MP Biomedicals, USA), following the manufacturer’s instructions. The integrity of the extracted DNA was verified by electrophoresis on a 1% agarose gel, and qualified samples were subsequently sent to Shanghai Lingen Biosequencing Company for sequencing. Both 16S rRNA gene amplicon sequencing and shotgun metagenomic sequencing were conducted on the Illumina NovaSeq 6000 platform. For 16S rRNA gene analysis, the V3-V4 hypervariable region was amplified via PCR using the universal primers 341F (5′-CCTAYGGGRBGCASCAG-3′) and 806R (5′-GGACTACHVGGGTWTCTAAT-3′). The resulting raw sequencing data were processed using the QIIME pipeline to generate an operational taxonomic unit (OTU) abundance table. Briefly, sequences were quality-filtered. Chimeras were detected and removed using USEARCH. Sequences were then clustered into OTUs at a 97% similarity threshold using the UCLUST algorithm. The most abundant sequence in each OTU was selected as the representative sequence. Taxonomy was assigned to each representative sequence using the RDP Classifier against the SILVA 132 database with a confidence threshold of 0.8. Finally, an OTU table was generated, which recorded the abundance of each OTU across all samples.

For metagenomic sequencing, a paired-end library was constructed from DNA fragments with a read length of 450 bp. Following library preparation, bridge PCR amplification was conducted, and the products were sequenced on the Illumina NovaSeq 6000 platform. The raw sequencing reads were first processed with the fastp software for quality control and adapter trimming. High-quality paired-end reads were then assembled *de novo* using MEGAHIT. Open reading frames were predicted from the assembled contigs using MetaGene. All predicted gene sequences from all samples were clustered using CD-HIT to construct a non-redundant gene catalog. High-quality reads from each sample were aligned against this non-redundant gene catalog using SOAPaligner with a 95% identity threshold, and the abundance of each gene was quantified. Representative sequences from the gene catalog were taxonomically and functionally annotated by aligning them against the National Center for Biotechnology Information (NCBI) Non-Redundant and Kyoto Encyclopedia of Genes and Genomes databases using BLASTP, with functional annotation for nitrogen metabolism pathways being of particular interest. All raw sequencing data generated in this study (16S rRNA amplicon and metagenomic data) have been submitted to the NCBI Sequence Read Archive (BioProject accession: PRJNA1431745). Due to constraints in sample processing and sequencing capacity, metagenomic analysis was performed only on spring and winter samples. Autumn samples were not included.

### Statistical analysis

The Kruskal-Wallis test combined with Dunn’s *post hoc* test (using the “stats” and “dunn.test” packages in R [v4.4.3]) was applied to assess seasonal differences in both the physicochemical factors and the alpha-diversity indices (Chao1, Shannon, and Simpson). Differences in microbial community structures across seasons were analyzed using non-metric multidimensional scaling (NMDS) and analysis of similarities (ANOSIM) based on the Bray-Curtis distance metric, implemented in the “vegan” package. A co-occurrence network between the top 50 nitrogen-metabolizing microorganisms and functional genes was constructed based on a strong Spearman correlation coefficient (|*r*| > 0.8, *P* < 0.05) (27). This network analysis was performed using R (v4.4.3) with the “psych” and “igraph” packages and visualized using Gephi (v0.10.1). RDA was performed using the “vegan” package to assess the relationships between environmental factors and both functional genes and microbial communities (28). Finally, a partial least squares path modeling (PLS-PM) approach was implemented using the “plspm” package to analyze the direct and indirect effects of seasonal changes, environmental factors, and microbial communities on nitrogen metabolism processes (29).

## RESULTS

### Physicochemical properties of water in the forebay of tributary pumping stations

**Fig 2:**
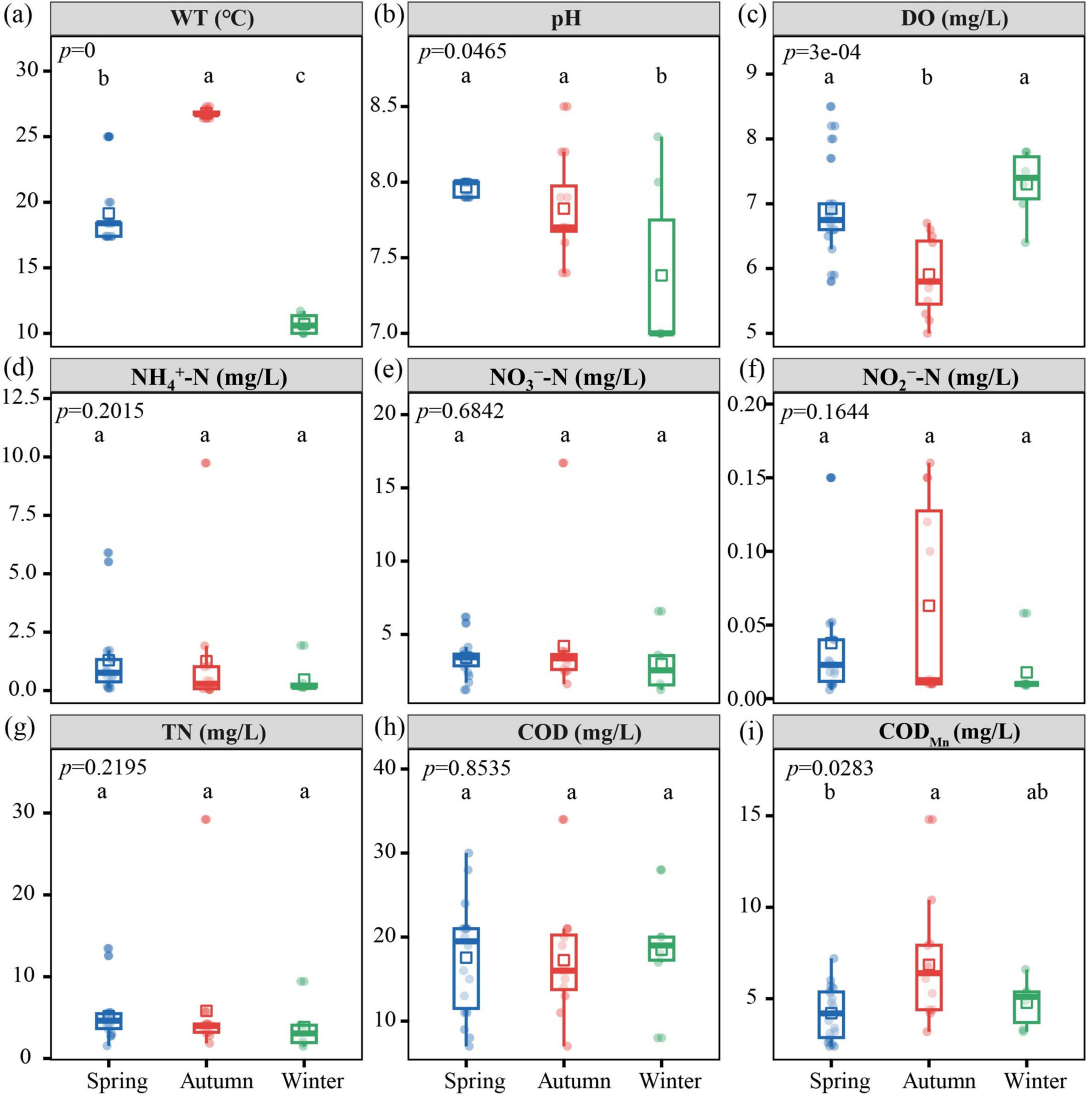
Seasonal variations of physicochemical indicators in the forebay of the tributary pumping station. (**a**) WT, (**b**) pH, (**c**) DO, (**d**) NH_4_^+^-N, (**e**) NO_3_^−^-N, (**f**) NO_2_^−^-N, (**g**) TN, (**h**) COD, and (**i**) COD_Mn_. Different lowercase letters (a, b, ab, c) indicate significant differences among seasons at *P* < 0.05. Groups sharing any common letter are not significantly different.

Changes in the physicochemical parameters of water samples collected from the tributary pumping station forebay in spring, autumn, and winter are shown in Fig. 2. The Kruskal-Wallis test results showed significant seasonal differences in WT overall (*P* < 0.001), with the mean WT values of 19.1°C, 26.8°C, and 10.7°C in spring, autumn, and winter, respectively (Fig. 2a). Among the remaining detection indicators, only pH (*P* < 0.05), DO (*P* < 0.01), and COD_Mn_ (*P* < 0.05) differed significantly between seasons. Specifically, the pH value in winter was significantly lower than in spring and autumn (Fig. 2b). DO was significantly lower in autumn than in spring and winter (Fig. 2c). COD_Mn_ in autumn was significantly higher than in spring and winter (Fig. 2i). The remaining indicators, including NH_4_^+^-N, NO_3_^−^-N, NO_2_^−^-N, TN, and COD, did not show significant seasonal differences throughout the observation period (Fig. 2d through h).

### Microbial community composition and diversity

**Fig 3:**
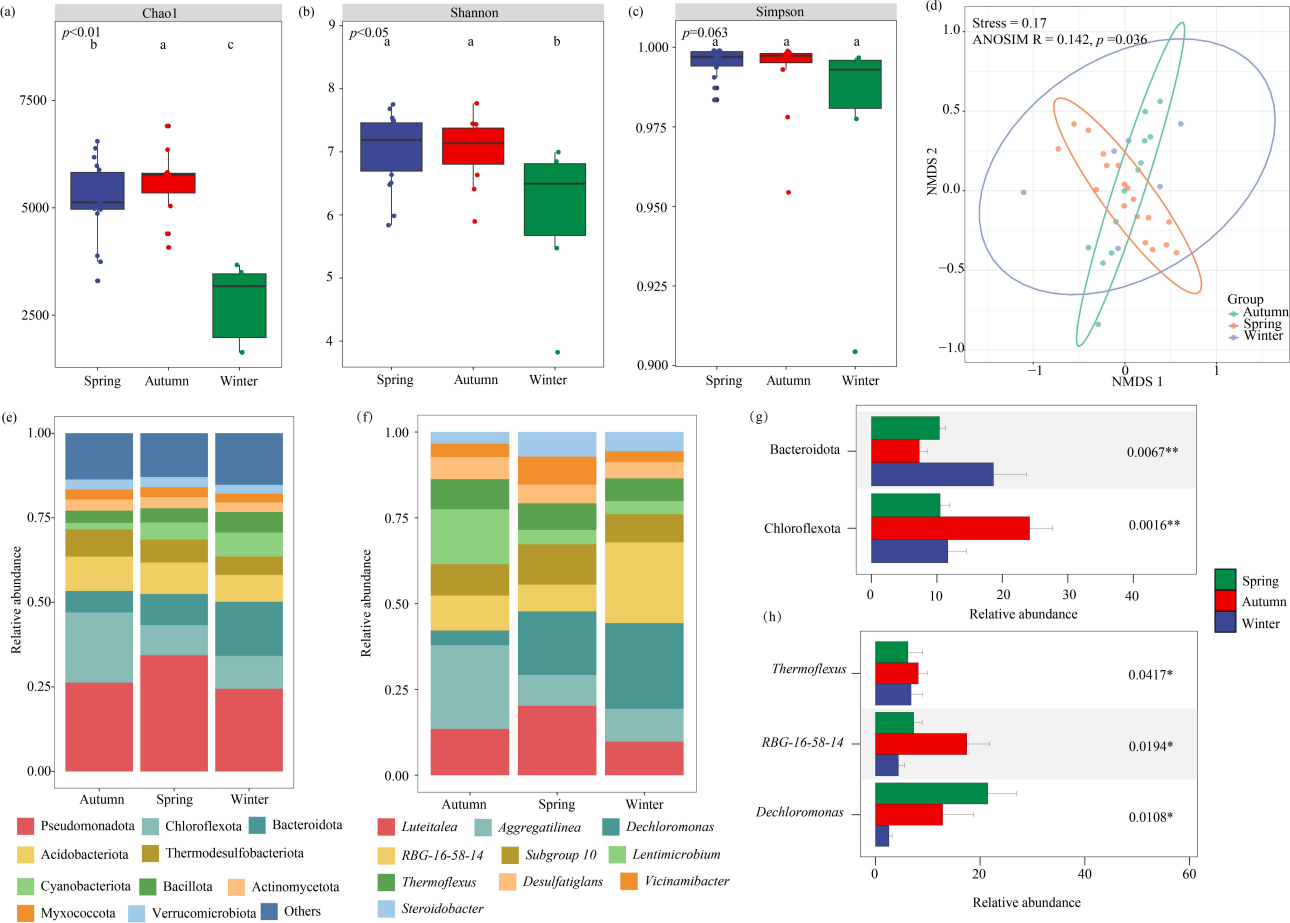
Seasonal variations in microbial community diversity and composition in the forebay of the tributary pumping station. (**a**) Chao1 index; (**b**) Shannon index; (**c**) Simpson index; (**d**) NMDS and ANOSIM analysis based on Bray-Curtis distance. (**e**) Microbial community composition at the phylum level. (**f**) Microbial composition at the genus level. (**g**) Phylum-level and (**h**) genus-level seasonal differences in microbial communities based on the Kruskal-Wallis test. *P < 0.05 and ***P < 0.01.

Based on OTU clustering analysis, the Chao1 index (reflecting species richness), Shannon index (representing species diversity), and Simpson index (indicating species dominance) were calculated. The results showed that both the Chao1 index (*P* < 0.01) and the Shannon index (*P* < 0.05) of the microbial community in the forebay of the tributary pumping station varied significantly across seasons (Fig. 3a and b). Specifically, the Chao1 index was significantly higher in spring and autumn than in winter, and the Shannon index was significantly higher in spring than in winter. In contrast, no significant seasonal difference was observed for the Simpson index (Fig. 3c). The NMDS ordination revealed a partial separation of microbial communities by season, with considerable overlap among samples from the same season (Fig. 3d). This visual pattern suggests that both temporal and spatial factors structure the communities. ANOSIM confirmed a statistically significant but moderate seasonal effect (*R* = 0.142, *P* = 0.036), demonstrating that seasonal variation was a detectable driver of community composition even when accounting for the influence of differing sampling sites.

Although the overall composition of the microbial communities was generally consistent across the three seasons, the relative abundances of these taxa varied considerably (Fig. 3e). The dominant bacterial phyla were Pseudomonadota, Chloroflexota, Bacteroidota, Acidobacteriota, and Thermodesulfobacteriota. The Kruskal-Wallis test (Fig. 3g) revealed that the abundance of Bacteroidota in winter was significantly higher than in spring and autumn (*P* < 0.01). Conversely, the relative abundance of Chloroflexota in autumn was significantly higher than in spring and winter. At the genus level (Fig. 3f and h), the top 10 most abundant genera were *Luteitalea*, *Aggregatilinea*, *Dechloromonas*, *RBG-16-58-14*, *Subgroup_10*, *Lentimicrobium*, *Thermoflexus*, *Desulfatiglans*, *Vicinamibacter*, and *Steroidobacter*. The relative abundance of *Dechloromonas* was significantly higher in spring than in autumn and winter, while *Thermoflexus* and *RBG-16-58-14* were significantly higher in autumn than in spring and winter (*P* < 0.05).

### Variations in key enzymes and functional genes

**Fig 4:**
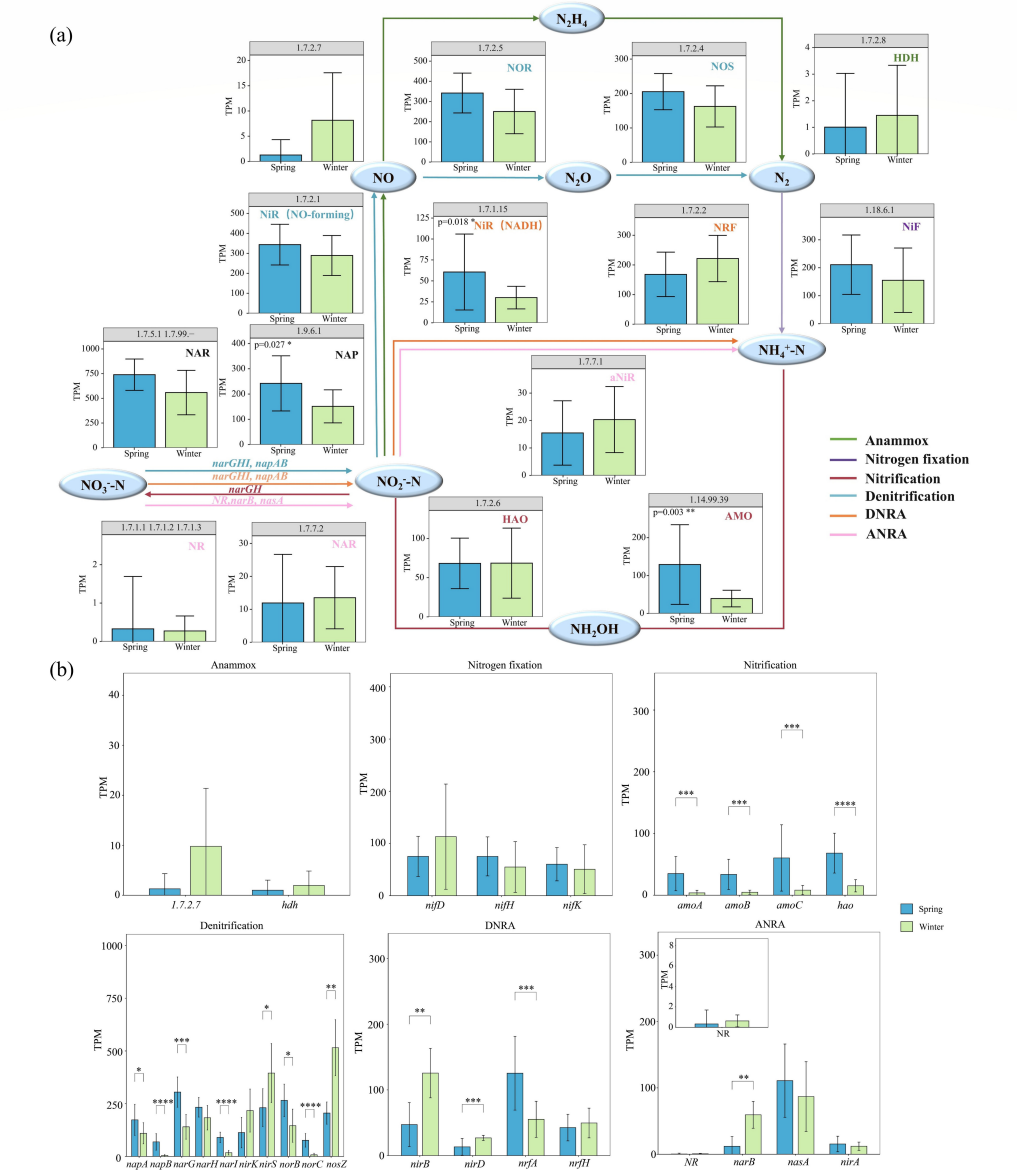
Seasonal variations in (**a**) enzymes and (**b**) functional genes related to nitrogen cycling in the forebay of the tributary pumping station. *P < 0.05, **P < 0.01, ***P < 0.001, and ****P < 0.0001.

Based on the NCycle database, we conducted a seasonal (spring vs winter) differential analysis of 27 nitrogen conversion-related functional genes and their corresponding enzymes. The analysis covered six major nitrogen metabolism pathways, including denitrification, nitrification, and nitrogen fixation (Fig. 4). The abundances of denitrification-related enzymes (NAR [EC 1.7.5.1, EC 1.7.99.-], NAP [EC 1.9.6.1], and Nir [EC 1.7.2.1]) and their key functional genes (*nosZ*, *nirS*, and *narG*) were the highest. This indicates that denitrification-related genes dominate in the sediment, suggesting that denitrification may be a key nitrogen transformation pathway in the forebay of the tributary pumping station. Furthermore, statistical analysis showed that the seasonal variations in the abundances of denitrification-related enzymes and key functional genes (*napAB*, *narGHI*, and *norBC*) were consistent, with significantly higher abundances observed in spring than in winter (*P* < 0.05).

In contrast, the abundances of anammox-related enzymes (EC 1.7.2.7, HDH [EC 1.7.2.8]) and functional genes (*hdh*), as well as ANRA-related enzymes (NR [EC 1.7.1.1, EC 1.7.1.2, EC 1.7.1.3], NAR [EC 1.7.7.2]) and functional genes (*NR*, *nirA*), were the lowest. These results suggest that the potential contribution of anammox and ANRA may be relatively minor in the forebay of the tributary pumping station. For nitrification, the abundances of the *amoABC* and *hao* genes were significantly higher in spring than in winter (*P* < 0.05), indicating greater nitrification potential during spring.

### Co-occurrence patterns between microbial communities and nitrogen cycling functional genes

**Fig 5:**
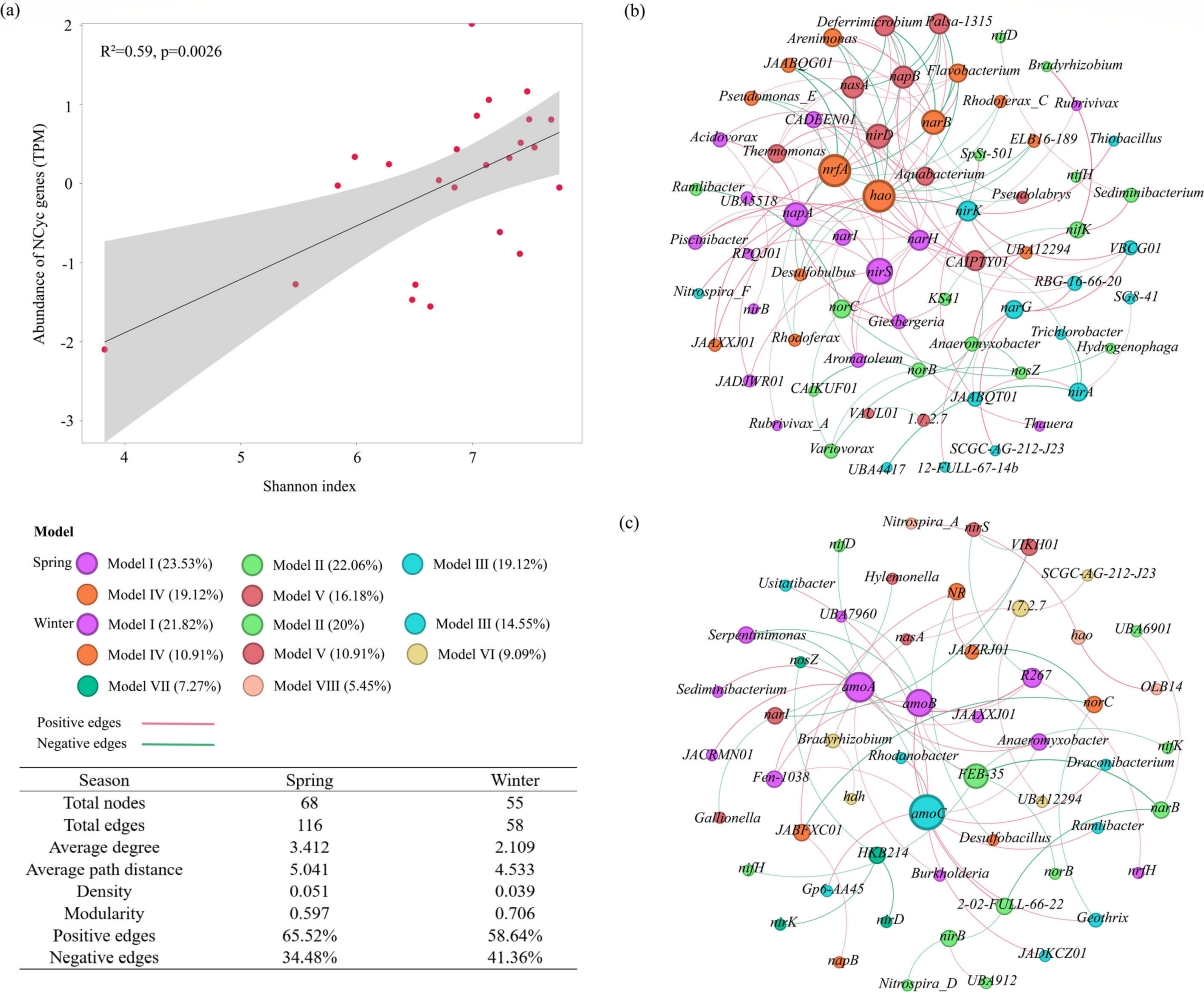
(**a**) Linear regression analysis illustrating the relationship between microbial community diversity and the abundance of nitrogen transformation functional genes. Co-occurrence networks between microbial genera and nitrogen transformation functional genes in (**b**) spring and (**c**) winter. Red lines indicate positive correlations, and green lines indicate negative correlations. The size of a node represents its degree (number of connections).

Linear regression analysis revealed a significant positive correlation between microbial community diversity and the abundance of nitrogen transformation functional genes (Fig. 5a*, R*^2^ = 0.59, *P* < 0.01). This strong positive relationship suggests that shifts in microbial community structure were closely associated with variations in nitrogen cycling metabolic potential. To further elucidate the interactions between nitrogen-metabolizing microorganisms and functional genes, a co-occurrence network was constructed between microbial genera involved in nitrogen metabolism and functional genes, based on taxonomic and genetic correspondence (Fig. 5b and c). The spring network consisted of 68 nodes and 116 edges (Fig. 5b), with an average weight of 3.142, an average path length of 5.041, a density of 0.051, and a proportion of positive correlations of 65.52%. In contrast, the winter network (Fig. 5c) contained fewer nodes (55) and edges (58) and exhibited lower values in average weight (2.109), average path length (4.533), and density (0.039). The proportion of positive correlations in the winter network was 58.64%. In both networks, positive correlations slightly outnumbered negative correlations. The higher connectivity metrics observed in the spring network, including greater numbers of nodes and edges, as well as higher average weight and density, indicate stronger associations between microbial genera and nitrogen-cycling functional genes during spring. This tighter coupling suggests enhanced potential interactions between microorganisms and nitrogen transformation processes in this season.

In the spring co-occurrence network, the nitrification gene (*hao*), the DNRA gene (*nrfA*), and the denitrification gene (*nirS*) exhibited extensive associations with microbial genera. Specifically, the primary potential hosts of the *hao* gene included *Arenimonas*, *Flavobacterium*, *JAABQG01*, and *JAAXXJ01*. The *nrfA* gene was primarily associated with *UBA12294*, *Flavobacterium*, *JAABQG01*, and *JAAXXJ01*. The main potential hosts for the *nirS* gene were *RPQJ01*, *CADEEN01*, and *Aquabacterium*. In the winter network, multiple nitrification genes (*amoA*, *amoB*, and *amoC*) showed broad connections with potential hosts. Key genera associated with these genes likely included *Anaeromyxobacter*, *Serpentinimonas*, and *Fen_1038*. Notably, despite its low abundance (Fig. 5b), the *NR* gene was present in the winter network and showed a significant positive correlation (*P* < 0.05) with the non-dominant genus *Desulfobacillus*. This suggests a possible cooperative relationship that may contribute to nitrogen cycling potential under environmental stress.

### Factors influencing microbial community and nitrogen transformation functional genes

**Fig 6:**
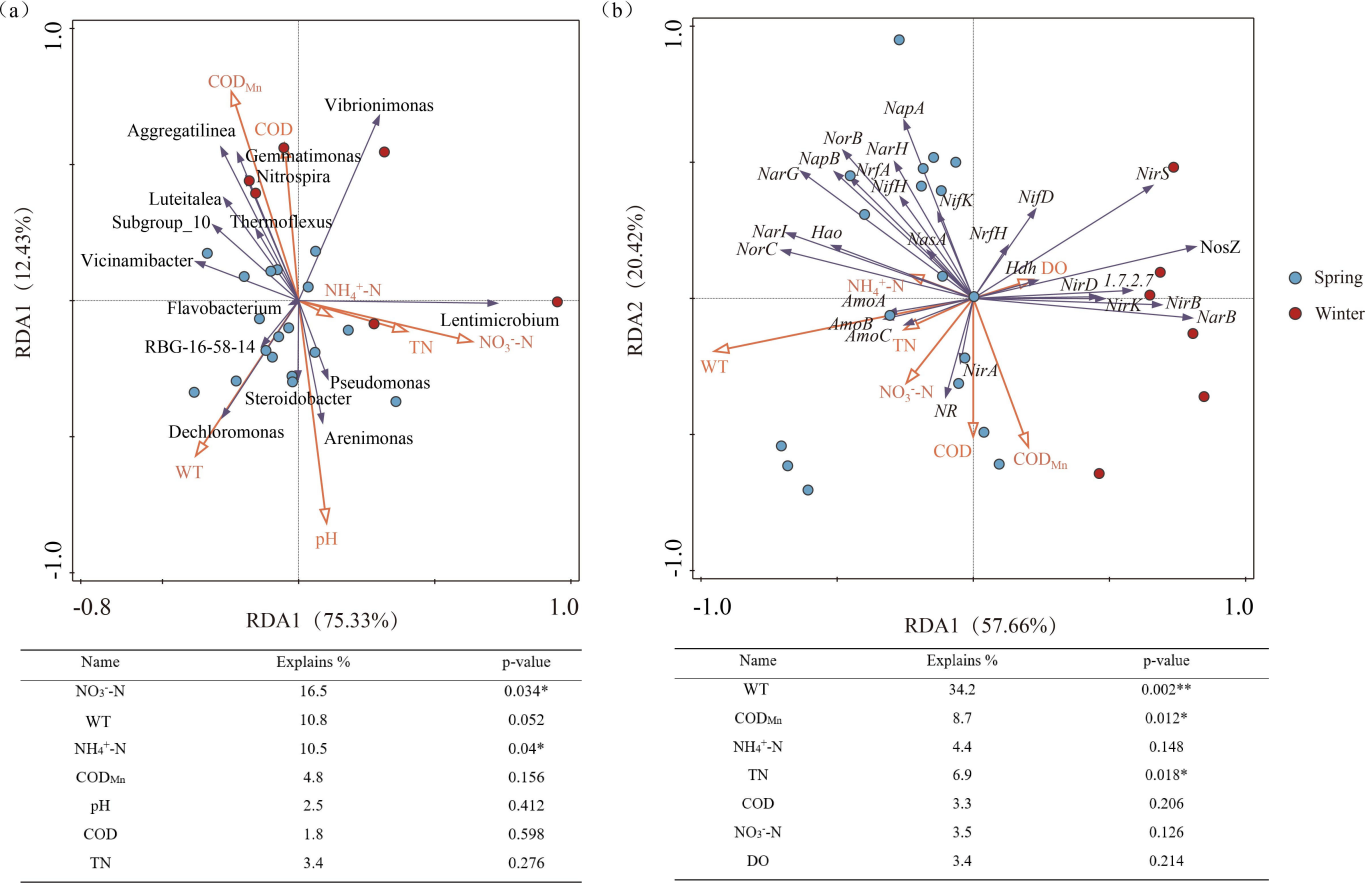
RDA showing the relationships between environmental factors and (**a**) microbial communities at the genus level and (**b**) nitrogen transformation functional genes. Orange lines represent environmental factors, while purple lines represent microbial genera or functional genes. The length of an arrow indicates the strength of its influence. *P < 0.05 and ***P < 0.01.

To identify the factors driving changes in microbial community composition and the distribution of nitrogen transformation functional genes, RDA was performed (Fig. 6). As shown in Fig. 6a, the RDA of microbial communities and environmental factors revealed that the first (RDA1) and second (RDA2) axes explained 75.33% and 12.43% of the variance, respectively, with a cumulative explanation of 87.76%. The correlation coefficients between the species-environment relationships and the two axes were 0.75 and 0.80, respectively, indicating that the ordination effectively captured the relationship between the microbial communities and environmental factors. Results from the Monte Carlo permutation test showed that NO_3_^−^-N, WT, and NH_4_^+^-N were the factors most strongly associated with microbial community distribution in the forebay of the tributary pumping station, explaining 16.5% (*P* < 0.05), 10.8% (*P* = 0.052), and 10.5% (*P* < 0.05) of the community variation, respectively.

As shown in Fig. 6b, RDA1 and RDA2 for functional genes explained 57.66% and 20.42% of the variance, respectively. The correlation coefficients between the gene-environment relationships and the axes were 0.90 and 0.80, respectively, demonstrating that the ordination reliably represents the relationship between nitrogen transformation functional genes and environmental factors. WT, COD_Mn_, and TN were identified as key environmental factors significantly associated with the distribution of these functional genes, accounting for 34.2% (*P* < 0.01), 8.7% (*P* < 0.05), and 6.9% (*P* < 0.05) of the variance explained, all of which reached significant or highly significant levels. Among these, WT had the highest explanation rate, indicating that it showed the strongest statistical association with the seasonal distribution of nitrogen conversion functional genes in the forebay of the tributary pumping station.

**Fig 7:**
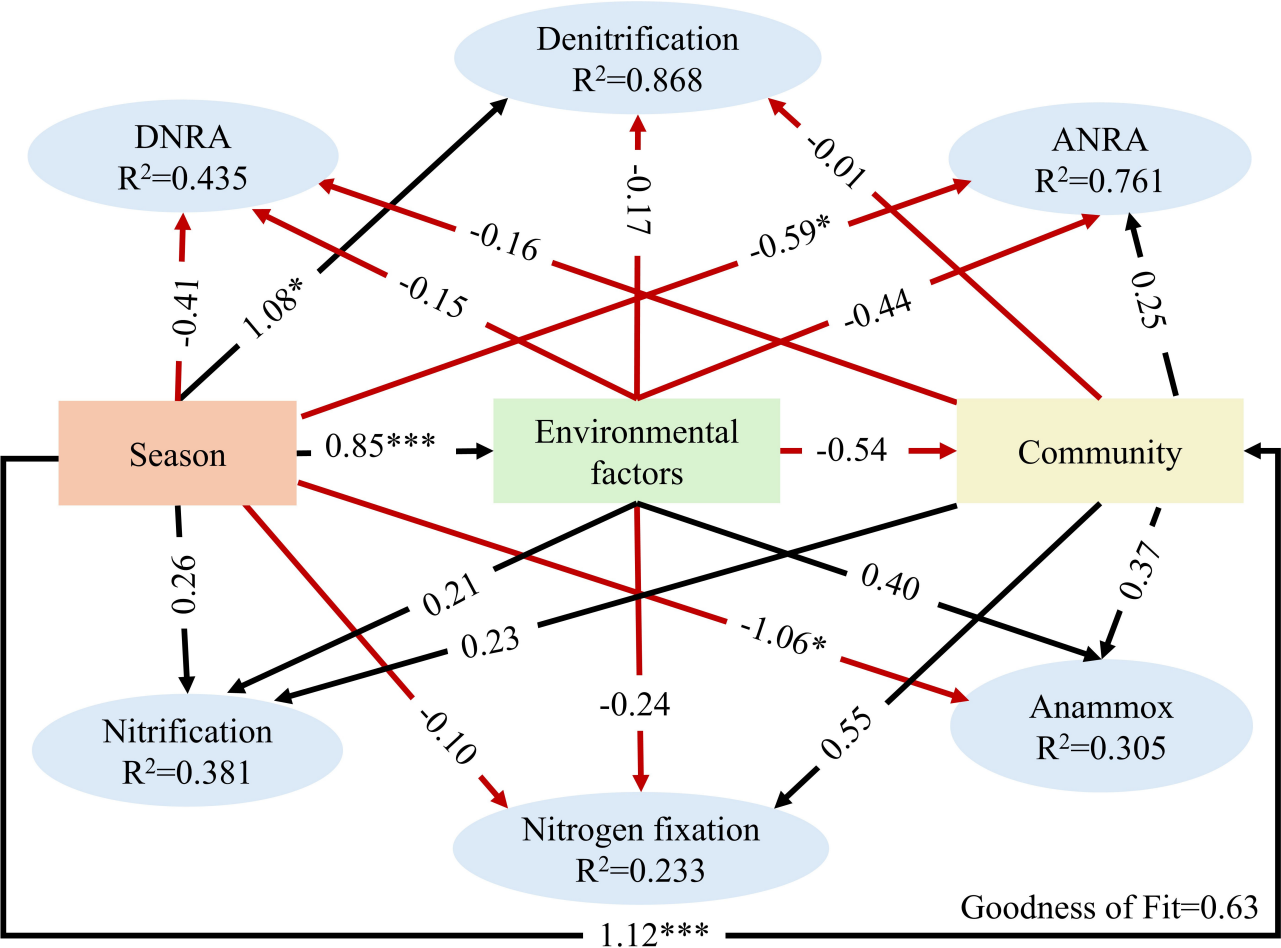
Results of the PLS-PM model illustrating the influencing factors and their standardized effects on nitrogen cycling processes. Black lines indicate positive effects, and red lines indicate negative effects. *P < 0.05, ***P < 0.001. Goodness of fit = 0.63.

Based on the PLS-PM model, which used data from spring and winter (due to the lack of functional gene data for autumn), the associations among seasonal variation, environmental factors, microbial community diversity, and nitrogen cycling-related gene abundance in the forebay of tributary pumping stations were further investigated (Fig. 7). The results identified seasonal variation as the factor most strongly associated with denitrification, ANRA, and anammox gene abundance (path coefficients: *β* = 1.08, *P* < 0.05; *β* = −0.59, *P* < 0.05; *β* = −1.06, *P* < 0.05), highlighting its central role in regulating nitrogen cycling in the forebay of tributary pumping stations. Furthermore, seasonal variation was significantly associated with environmental factors and microbial community diversity (*β* = 0.85, *P* < 0.0001; *β* = 1.12, *P* < 0.0001). It is noteworthy that the direct associations of environmental factors and microbial community diversity with nitrogen cycling gene abundance did not reach statistical significance. Collectively, the PLS-PM results indicate that seasonal variation was the factor most strongly associated with changes in nitrogen-cycling gene abundance.

## DISCUSSION

### Seasonal dynamics of environmental parameters

The physicochemical characteristics of the water in the forebay of tributary pumping stations exhibited clear seasonal variations, reflecting the strong influence of regional hydrological and climatic conditions. WT showed the most pronounced seasonal variation, consistent with the pattern typical of subtropical monsoon climates. Seasonal differences in DO, COD_Mn_, and pH indicate variations in oxygen availability and organic matter loading, which together influence microbial metabolic processes. The relatively low DO and high COD_Mn_ observed in autumn suggest enhanced organic loading and oxygen depletion during this period. These patterns may be associated with seasonal hydrological dynamics and increased inputs of organic matter and nutrients from surrounding catchments (30). These environmental fluctuations likely modify environmental filtering and resource availability, thereby driving microbial community turnover and functional differentiation across seasons.

### Seasonal shifts in microbial community composition and diversity

The observed variations in microbial richness and diversity indicate that seasonal environmental changes significantly reshape microbial community structure in the forebay ecosystem. Higher richness and diversity in spring likely result from optimal temperature, abundant organic carbon and nutrient resources, and relatively stable hydrodynamic conditions. These conditions support a broad range of microbial taxa with distinct metabolic strategies, thereby contributing to higher community diversity. In contrast, the reduced diversity in winter suggests that low temperature and limited nutrient supply selectively favor cold-adapted taxa and reduce community evenness (31). Although the presence of dominant phyla such as Pseudomonadota, Chloroflexota, and Bacteroidota remained stable across seasons, their relative abundances varied substantially. This pattern suggests that seasonal environmental changes primarily affect the relative abundances of different microbial groups, while the overall community structure at the phylum level remains stable. The NMDS and ANOSIM analyses further demonstrated that while seasonal differences were statistically significant, they were moderate (*R* = 0.142, *P* = 0.036), implying that spatial heterogeneity among sampling sites also contributed to community differentiation.

At the genus level, dominant genera, including *Luteitalea*, *Dechloromonas*, and *Lentimicrobium*, play key roles in nitrogen cycling and are widely involved in nitrogen metabolism. *Dechloromonas*, for example, utilizes nitrate as an electron acceptor and reduces it to nitrogen gas through denitrification. This genus plays a vital role in both wastewater treatment systems and natural environments, helping to remove nitrate and mitigate eutrophication risks (32, 33). The strong representation of *Dechloromonas* in spring samples likely reflects favorable conditions for denitrification, including sufficient organic carbon and nitrate availability, together with relatively low oxygen levels. These findings suggest that shifts in the dominance of functionally versatile taxa underpin the observed seasonal variability in nitrogen transformation potential.

### Functional gene dynamics and nitrogen transformation pathways

Seasonal changes in nitrogen transformation functional genes likely reflect the effects of temperature and substrate availability on nitrogen metabolism. The higher abundance of denitrification- and nitrification-related genes (such as *narG*, *nirS*, and *amoABC*) in spring suggests greater potential for microbial nitrogen transformations under warmer conditions. Denitrification-related genes dominate in the sediment metagenome, indicating that denitrification may be a key nitrogen transformation pathway in the forebay. This aligns with previous studies showing that heterotrophic denitrifiers are favored in nutrient-rich, organic-loaded aquatic systems (34). In contrast, the reduced abundance of these genes during winter suggests metabolic slowdown and a shift toward nitrogen accumulation, likely due to low temperatures and limited electron donors. The weak signal of anammox- and ANRA-related genes further implies that these processes played a minor role in the studied system. Collectively, these results suggest that denitrification potential was relatively higher in the forebay ecosystem, with nitrification likely contributing to local nitrate availability, potentially influenced by multiple nitrogen sources, while anammox and assimilatory reduction appeared to play comparatively minor roles in the overall nitrogen transformation balance.

### Co-occurrence network complexity and ecological stability

The co-occurrence network analysis provides additional insights into how microbial interactions respond to seasonal transitions. The spring network displayed higher connectivity, density, and average weight than the winter network, suggesting that microbial associations were more complex under favorable environmental conditions, which may imply greater potential for functional interactions. Predominantly positive correlations suggest that cooperative and syntrophic relationships, rather than competition, are the dominant interaction modes during active nitrogen cycling periods.

Such network complexity is often linked to greater ecosystem stability and functional resilience (35). In the present study, the tighter coupling between denitrification genes (e.g., *nirS* and *norBC*) and their potential host genera during spring may contribute to more robust nitrogen cycling potential. Conversely, the simplified winter network may reflect weakened microbial interactions and reduced system resilience under low-temperature stress. These seasonal shifts in microbial co-occurrence patterns suggest a dynamic balance between community cooperation and environmental constraints in regulating nitrogen-cycling efficiency.

### Environmental drivers and integrative regulation of nitrogen cycling

RDA identified WT as the environmental factor most strongly associated with both microbial community composition and nitrogen functional gene distribution. WT is widely recognized as influencing enzymatic reaction rates and DO dynamics in aquatic systems. The strong statistical associations observed here suggest that WT may play an important role in shaping nitrogen transformation processes in the forebay sediments. The influence of temperature on denitrification aligns with established knowledge from diverse aquatic systems, confirming it as a pivotal and widespread mechanism for regulating nitrogen loads (36, 37). COD_Mn_ and TN were also key explanatory variables, highlighting the potential role of organic carbon and nitrogen substrate supply in shaping microbial metabolic networks.

The PLS-PM results further revealed that seasonal variation was significantly associated with nitrogen cycling-related gene abundance, as well as with environmental gradients and microbial diversity. The non-significant effects of environmental variables and community diversity on gene abundance may suggest that community structure may not directly determine ecosystem functioning, possibly due to asynchronous or time-lagged responses.

Taken together, these findings suggest that seasonal variation may be associated with changes in nitrogen cycling-related genes in pumping station forebays, potentially through its influence on WT, oxygen availability, and organic matter availability, which may, in turn, shape microbial communities and their functional linkages. These patterns provide insights into the sensitivity of nitrogen cycling potential to seasonal environmental changes and could inform the optimization of operation schedules and pollution control strategies in urban tributary systems.

### Limitations and future perspectives

This study has two primary limitations. First, the absence of summer sampling and autumn metagenomic data prevents a comprehensive analysis of the microbial nitrogen cycle across the entire annual cycle. Second, this study assessed genetic potential based on functional gene abundance, rather than measuring actual process rates (e.g., denitrification and nitrification). Therefore, our interpretations, including the inferred predominance of denitrification, should be regarded as inferences based on genetic potential rather than direct evidence of process dominance. Third, the use of different sampling sites across seasons means that spatial heterogeneity might partially confound the temporal interpretation. Future research should therefore implement year-round sampling at consistent locations, combined with direct measurements of nitrogen transformation rates, to fully resolve the seasonal succession of microbial communities and their functional traits.

### Conclusion

This study systematically investigated the water quality characteristics, microbial community structure, nitrogen cycling-related genes, and their potential driving factors in the forebay ecosystem of a tributary pumping station across seasons. The results revealed significant seasonal variations in WT, pH, DO, and COD_Mn_, whereas nitrogen nutrient indicators remained largely stable. Seasonal variation significantly influenced the diversity and composition of the microbial community, with higher diversity observed in spring compared to winter. Denitrification-related genes dominated in the sediment, with significantly higher abundance in spring than in winter, suggesting this pathway may play a key role in nitrogen cycling. In contrast, anammox- and ANRA-related genes exhibited low abundance, implying their potential contribution is relatively minor. Microbial community diversity showed a significant positive correlation with the abundance of nitrogen transformation genes. The microbial-gene co-occurrence network exhibited greater complexity and connectivity in spring, suggesting more complex potential interactions that may contribute to the stability of these interaction networks during this season. NO_3_^−^-N, WT, and NH_4_^+^-N were the factors most strongly correlated with microbial community structure, while WT, COD_Mn_, and TN were significantly associated with the distribution of nitrogen functional genes. Furthermore, seasonal variation exerted significant effects on the abundance of genes related to denitrification, ANRA, and anammox (with standardized path coefficients of 1.08, −0.59, and −1.06, respectively). Overall, this study provides insights into the seasonal dynamics of microbial communities and nitrogen transformation potential in pumping station forebays, highlighting the influence of environmental factors such as temperature and nutrient availability. These findings may contribute to improving water quality management and optimizing pumping station operation strategies in urban tributary systems.

## Data Availability

The raw sequence data from this study have been deposited in the National Center for Biotechnology Information (NCBI) Sequence Read Archive (SRA) under BioProject accession number PRJNA1431745.
